# “Targeted Sequencing by Gene Synteny,” a New Strategy for Polyploid Species: Sequencing and Physical Structure of a Complex Sugarcane Region

**DOI:** 10.3389/fpls.2018.00397

**Published:** 2018-03-28

**Authors:** Melina C. Mancini, Claudio B. Cardoso-Silva, Danilo A. Sforça, Anete Pereira de Souza

**Affiliations:** ^1^Center for Molecular Biology and Genetic Engineering, University of Campinas, Campinas, Brazil; ^2^Departament Plant Biology, Biology Institute, University of Campinas, Campinas, Brazil

**Keywords:** polyploid, physical map, BAC, *Saccharum hybridum*, sugar accumulation, complex genome

## Abstract

Sugarcane exhibits a complex genome mainly due to its aneuploid nature and high ploidy level, and sequencing of its genome poses a great challenge. Closely related species with well-assembled and annotated genomes can be used to help assemble complex genomes. Here, a stable quantitative trait locus (QTL) related to sugar accumulation in sorghum was successfully transferred to the sugarcane genome. Gene sequences related to this QTL were identified *in silico* from sugarcane transcriptome data, and molecular markers based on these sequences were developed to select bacterial artificial chromosome (BAC) clones from the sugarcane variety SP80-3280. Sixty-eight BAC clones containing at least two gene sequences associated with the sorghum QTL were sequenced using Pacific Biosciences (PacBio) technology. Twenty BAC sequences were found to be related to the syntenic region, of which nine were sufficient to represent this region. The strategy we propose is called “targeted sequencing by gene synteny,” which is a simpler approach to understanding the genome structure of complex genomic regions associated with traits of interest.

## Introduction

When no previously reported genome is available, genome reconstruction is based on a *de novo* assembly strategy (based on sequence read overlap). This task becomes more complicated when an organism has a large genome with highly abundant repetitive elements.

Polyploid species account for approximately one-third of all plants (Wood et al., 2009), many of which are crops with great economic importance, such as wheat, cotton, potato and sugarcane. Sugarcane (*Saccharum* sp.) is the crop with the most complex genome structure because modern sugarcane varieties are derived from interspecific hybridization between *Saccharum officinarum* (basic chromosome number: *x* = 10; 2*n* = 8*x* = 80) and *Saccharum spontaneum* (basic chromosome number: *x* = 8; 2*n* = 5*x* = 40 to 16*x* = 128). The resulting hybrids are highly polyploid and aneuploid, with chromosome numbers ranging from 80 to 128 (D'Hont et al., 1998; Irvine, 1999; Grivet and Arruda, 2001) and an estimated whole-genome size of 10 Gb (D'Hont and Glaszmann, 2001). Previous studies have shown that ~50% of the sugarcane genome is composed of repetitive sequences (Figueira et al., 2012; Kim et al., 2013; de Setta et al., 2014).

Several studies using bacterial artificial chromosomes (BACs), involving either individual BAC assembly (de Setta et al., 2014; Vilela et al., 2017) or pooled strategies (Okura et al., 2016; Visendi et al., 2016), have been reported. In both cases, the applied sequencing strategies are based on the selection of non-overlapping BAC clones. Moreover, a draft sugarcane genome based on whole-genome shotgun sequencing of the SP80-3280 hybrid has been published (Riaño-Pachón and Mattiello, 2017). However, the main problem lies in reconstructing large and complex regions of the genome to represent a specific region of interest. In the present study, the synteny between related species, sorghum (*Sorghum bicolor*) and sugarcane (*Saccharum* sp.), was explored. Among the grasses that have been studied to date, sorghum is considered the closest ancestor of the *Saccharum* complex. Sugarcane and sorghum shared a common ancestor ~5 million years ago (Paterson et al., 2004), while sugarcane and its sister genus *Miscanthus* share a common ancestor separated by ~3.8–4.6 million years (Kim et al., 2014). Using the sorghum genome as a reference for annotation is advantageous because it has been completely sequenced and annotated (Paterson et al., 2009). Additionally, some sorghum varieties, referred to as sweet sorghum [*Sorghum bicolor* (L.) Moench], are capable of storing sugar in their stems (Vietor and Miller, 1990). Here, we propose the “targeted sequencing by gene synteny” strategy of sugarcane BAC selection for the reconstruction of a complex sugarcane genome region linked to a quantitative trait locus (QTL) mapped for sugar accumulation (Brix) (Murray et al., 2008) at a specific position on sorghum chromosome 3 (SB-03), based on the high synteny between the sugarcane and sorghum genomes.

## Materials and methods

### *In silico* data sources (sorghum and sugarcane)

A QTL for Brix was chosen from a study by Murray et al. (2008), which identified the QTL in the SB-03 genome (see Data Sheet S1 topic “*In silico* data sources”). The sequences of each molecular marker in this region were employed to locate the chromosome position using the sorghum genome v3.1, available on the Phytozome 12.0 database (http://www.phytozome.net/), as a reference. An alignment between sorghum genes and sugarcane transcripts (Cardoso-Silva et al., 2014) was performed through a BLASTn analysis with a cutoff *E* < 1e10. In this step, we selected the best hit for each query alignment (Table S1). We designed primer pairs flanking single and conserved exons predicted by alignments between sugarcane and sorghum genes (Table S2).

### BAC library screening, BAC pooling, and sequencing

BAC clones from the Brazilian hybrid sugarcane cultivar SP80-3280 that contained the specific selected genes were chosen through screening of 3D pools (see Data Sheet S1 topic “BAC library screening”). Positive BAC clones containing the same gene were sequenced in different pools to avoid casual overlap of BACs containing homeologous regions. A total of 68 BAC clones were arranged in nine sequencing pools. SMRTbell libraries for sequencing were prepared using the 20 kb procedure according to the Pacific Biosciences (PacBio) protocol, and sequencing was performed at the Arizona Genomics Institute (AGI; Tucson, USA) using a SMRT DNA sequencing system available from PacBio.

### Read trimming and BAC assembly

The PacBio long reads were masked for vector sequences (*pIndigo*BAC5) using cross_match (-minmatch 10 -minscore 20 -screen), and *E. coli* str. K-12 genomic DNA was removed. *De novo* assembly was performed with the hierarchical assembly pipeline PBcR (the PacBio Corrected Reads Pipeline), implemented as part of wgs-assembler v8.3rc2 (Berlin et al., 2015) and Celera Assembler (Myers et al., 2000). The minimum length of the sequences for correction was set to 500 bp, and the number of partitions for consensus was set to 200. The contigs obtained with the assembler were subjected to error correction by remapping the reads with pbalign (v0.2). The PacBio reads were aligned using the BLASR algorithm (Chaisson and Tesler, 2012), and we performed assembly polishing with the Quiver tool (Chin et al., 2013). See Data Sheet S1 topic “BAC assembly” for more details.

### BAC annotation and synteny analysis

The BAC sequences were annotated in two steps. First, we used a method to predict long terminal repeat transposons (LTRs) via LTR_FINDER (Xu and Wang, 2007). Homology-based repeat analysis was performed to identify transposable elements (TEs) against Poaceae TEs available in the Repbase database (Kohany et al., 2006) via CENSOR. Second, genes were manually predicted using the sorghum genome annotation as a reference. All annotations were manually curated using Artemis: Genome Browser and Annotation Tools (Rutherford et al., 2000). Additionally, sugarcane CDS genes were translated into protein and were aligned by BLASTp (cutoff *E* < 1e-10) against the sorghum, maize, and rice proteomes obtained from the Phytozome 12.0 database.

## Results

### *In silico* data sources (sorghum and sugarcane)

Sequence-based marker information related to the QTL for Brix (Murray et al., 2008) was employed for linkage to the physical location on SB-03 (from Sb3:55,265 kb to Sb3:55,952 kb; sorghum genome v3.1 available on Phytozome 12.0 database), comprising ~700 kb in length (Data Sheet S2). A total of 61 predicted genes were found within this region in the sorghum genome, and these genes were used for alignment against the sugarcane transcriptome described by Cardoso-Silva et al. (2014). Fifty-three sorghum genes showed high similarity to sugarcane transcripts (Table S1). One primer pair for each of the 53 selected genes was synthesized using the sugarcane transcriptome as a template (Table S2).

### BAC library screening, sequencing, and assembly

The primers showing good amplification were employed in the 3D pool screening method. To increase the chance of recovering the homologous region in the sugarcane genome, BAC clones were only selected if they had at least two positive markers. Based on this strategy, a total of 68 BAC clones were identified, pooled, and further sequenced (see Data Sheet S1 topic “BAC library screening”).

Thus, a total of 1,660,342 trimmed long reads were obtained; the number of reads per pool ranged from 139,394 (Pool 03) to 237,520 (Pool 06), with a mean of 184,482 long reads per pool (Table 1). The percentage of reads that represented contamination by the *E. coli* genome was 8.25% on average, ranging from 5% (Pool 01) to 13% (Pool 03).

**Table 1:** Statistical summary of the sequencing (via PacBio) and assembly of the sugarcane BAC pools from the Brazilian hybrid sugarcane cultivar SP80-3280.

**PacBio sequencing**				**Celera assembly**					
**Name**	**N. BACs**	**Trimmed reads**	***E. coli* %**	**Contigs**	**Longest contig**	**Smallest contig**	**Contig total length**	**N50**	**GC (%)**
Pool 01	4	178,758	5	16	143,471	9,202	582,340	62,347	43.77
Pool 02	8	202,770	7	17	134,154	8,615	1,034,115	109,126	45.08
Pool 03	8	139,394	13	25	142,211	8,101	800,349	54,726	45.45
Pool 04	8	206,601	8	27	122,448	9,303	882,224	41,554	44.06
Pool 05	8	189,764	9.6	16	175,157	8,050	1,186,577	132,868	45.28
Pool 06	8	237,520	9	21	168,704	8,289	920,150	86,198	44.75
Pool 07	8	143,827	6.8	19	164,848	10,668	1,140,957	128,641	45.41
Pool 08	8	186,873	7.4	19	143,661	10,202	1,129,862	108,955	44.41
Pool 09	8	174,835	8.4	20	187,285	10,664	1,261,846	99,030	44.43
Total	68	1,660,342	–	180	–	–	8,938,420	–	–

Assembly was performed individually for each pool. The number of contigs that originated from the pools ranged from 16 (Pools 01 and 05) to 27 (Pool 04), with a mean number of contigs of ~20. A total of 180 contigs were obtained through Celera assembly, with sizes ranging from 187,285 kb (Pool 09) to 8,050 kb (Pool 05). The total length of all the assembled contigs was 8.94 Mb, with an N50 contig length of 91.5 kb and a GC content of 44.74%. The N50 value was higher than that obtained during wheat BAC sequencing using only long reads generated by PacBio, which exhibited a mean N50 of 80 kb (Visendi et al., 2016).

**Figure 1:**
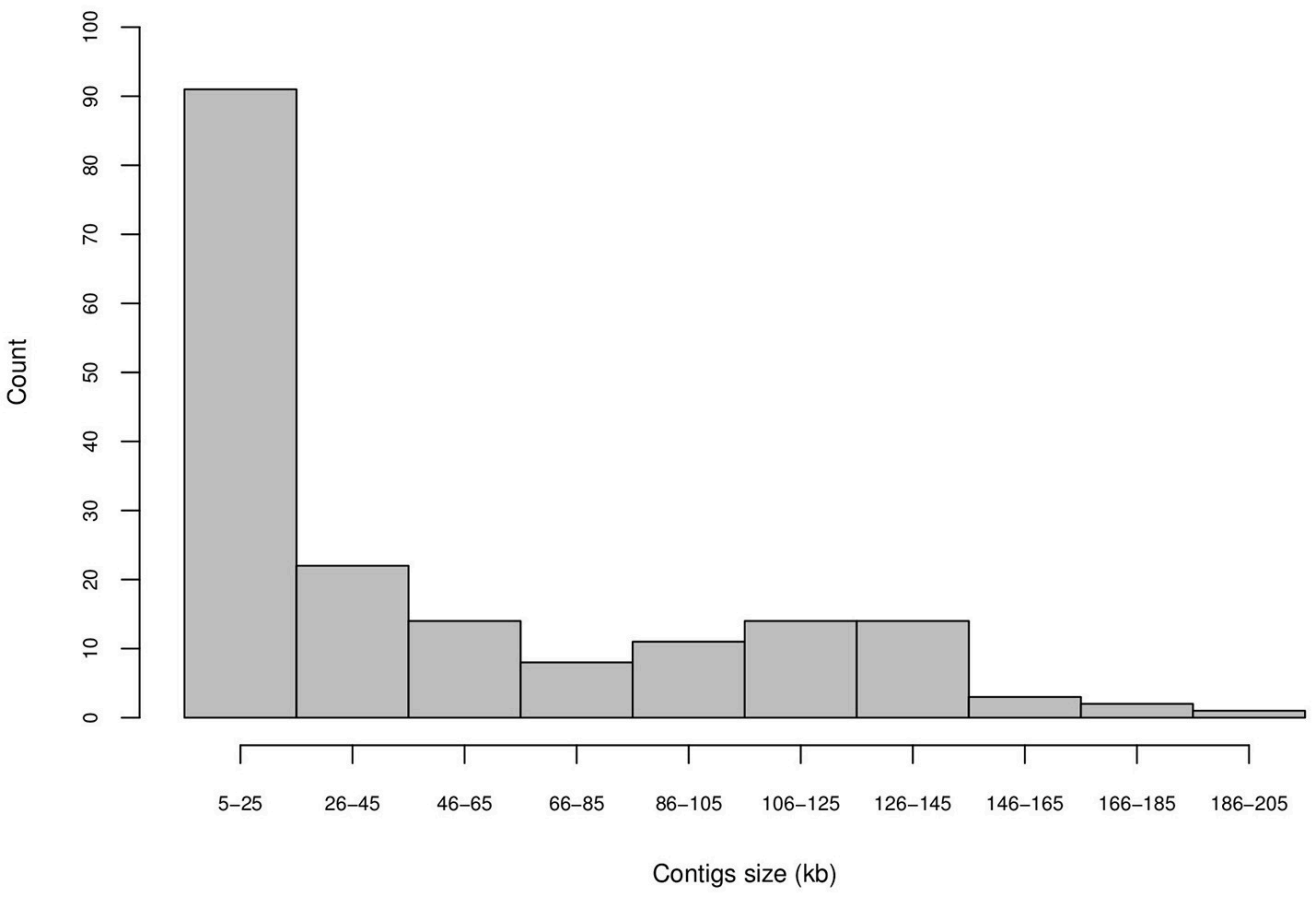
Length distributions of the 180 sugarcane contigs obtained by the assembly of sugarcane BAC pools from the Brazilian hybrid sugarcane cultivar SP80-3280.

Most of the assembled contigs (112 contigs, 62.2% of the total) exhibited lengths smaller than 50 kb (Figure S2) and/or showed low coverage assembly (Figure S1); these contigs were not considered in further analyses. However, 68 of the assembled contigs exhibited suitable lengths and high coverage (Figure 1).

### BAC annotation

A total of 68 BACs representing the longest contigs with high coverage (Figures S3, S4) were selected for gene annotation and repetitive element screening. Approximately 51% of the assembled and annotated BACs were identified as repetitive elements, including 41% of long terminal repeat retrotransposons (LTR), 8% of DNA transposons and 2% of non-LTRs. Within the LTRs, the most common groups were *Gypsy* and *Copia*, representing 58 and 42% of the total, respectively (Table S3).

A total of 253 complete coding genes were predicted in 55 sugarcane BAC sequences using the sorghum genome as a reference, 211 of which were unique genes, with the number of genes ranging from one to 13, yielding a gene density of one gene per 23.6 kb (Table S4). A total of 245 and 243 of these genes were shared with rice and maize, respectively. Additionally, 134 mobile elements inserted within genes were identified, with 69 genes containing inserted mobile elements ranging from 146 bp (Stowaway) to 11,800 bp (LTR/*Copia*) in size.

### Corresponding region of the sorghum QTL and synteny analyses

**Figure 2:**
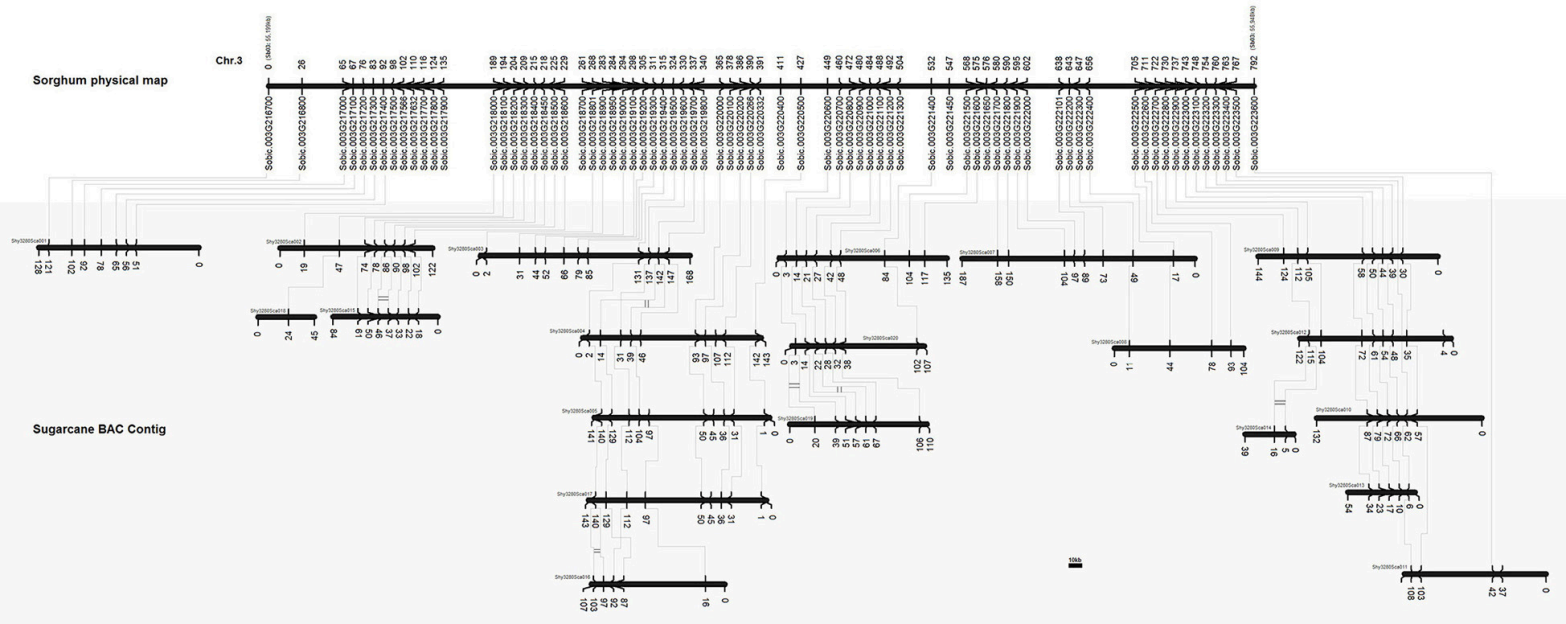
Physical representation of a specific sorghum genome region (Chr3) containing a QTL for sugar accumulation and sugarcane BAC sequences (shaded gray box) from the Brazilian hybrid sugarcane cultivar SP80-3280. The sorghum gene annotation and position (starting in zero kb, representing the beginning of the specific region) were included. The synteny between sorghum and sugarcane genes is represented as is the genomic organization, including the homeologous BAC sequences and tandem duplication in sugarcane genes (double line connection).

Based on the analysis of the physical map, it was possible to define the homeologous chromosomes and gene duplications. In total, 20 BAC sequences were successfully mapped to the corresponding sorghum gene position (Figure 2). A total of 74 genes were observed in this interval in sorghum, while 59 were identified in sugarcane.

**Figure 3:**
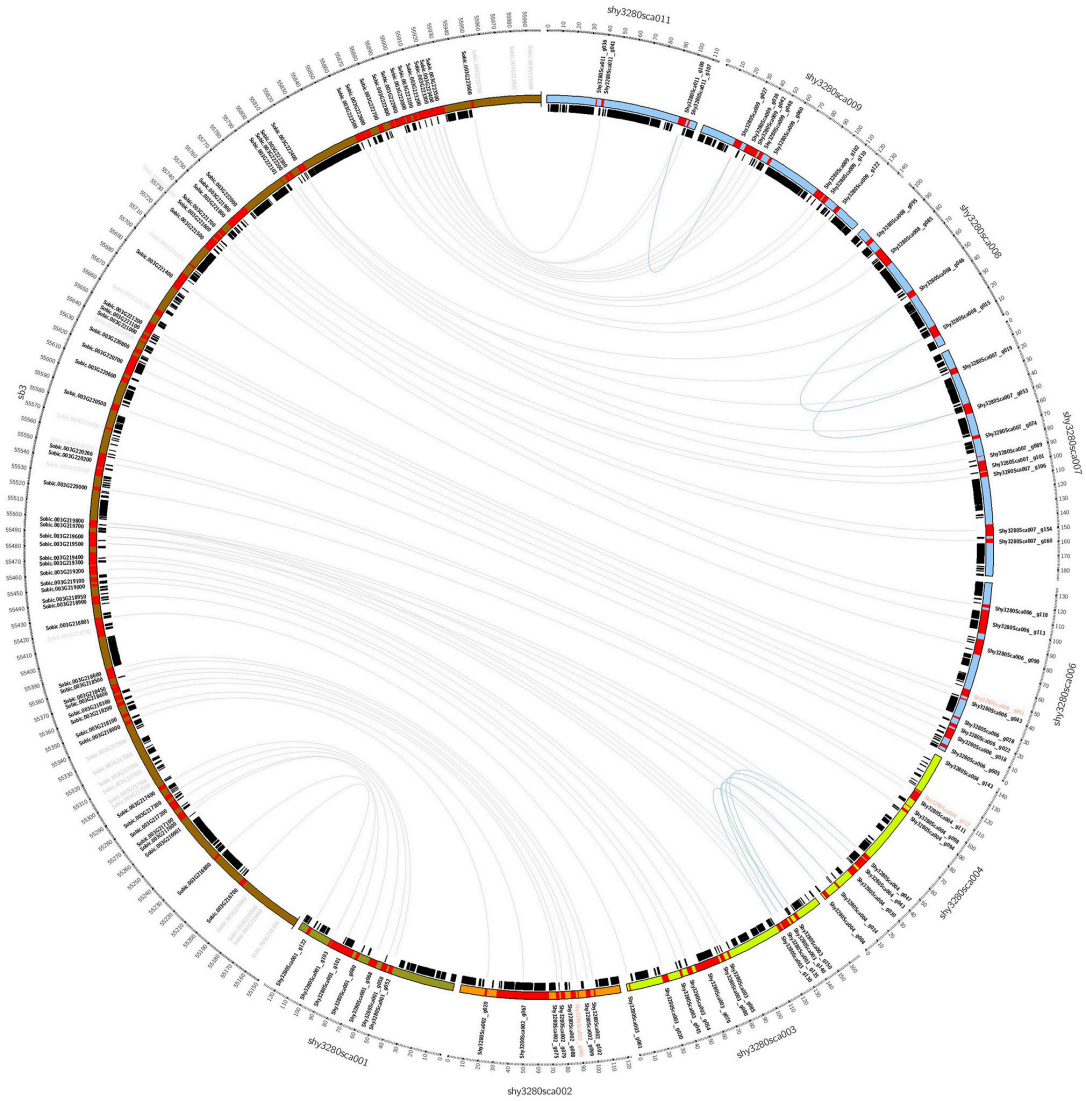
Structural organization relationship among the genes on chromosome 03 in *Sorghum bicolor* (golden brown) and the four blocks of BAC sequences in sugarcane based on synteny. The sugarcane blocks are represented by different colors: green (Shy3280Sca001), orange (Shy3280Sca002), light green (Shy3280Sca003 and Shy3280Sca004), and light blue (Shy3280Sca006, Shy3280Sca007, Shy3280Sca008, Shy3280Sca009, and Shy3280Sca011).

Using the genes annotated in sugarcane as a reference, a total sequence length of 1.25 Mb was necessary to partially cover the target region in SB-03, which was represented by nine BAC sequences (Figure 3) divided into four syntenic blocks. There were three gaps found among the four sugarcane syntenic blocks. In two situations, we found sorghum genes without a corresponding BAC sequence between: shy3280sca001 and shy3280sca002 (Sobic003G217500 to Sobic003G217900) and shy3280sca002 and shy3280sca003 (Sobic003G218700); while between shy3280sca004 and shy3280sca006, there were two consecutive sorghum genes that had different BAC sequences.

## Discussion

The regions that control economically important traits are often influenced by several genes, and QTL mapping is typically used to determine the genomic position of *loci* that phenotypically influence a desired trait. In sugarcane, these effects are usually low. Genomic characteristics such as a high ploidy level show complex allele dosage and distribution on different homeologous chromosomes, which could explain the lower contribution of individual genes and/or alleles. Therefore, most studies have mapped single allelic doses. The percentage of phenotypic variance explained for various traits ranges from 0.069% (Costa et al., 2016) to 16.2% (Ming et al., 2002). However, such effects are more pronounced in sorghum, ranging from 7.7% (Shiringani et al., 2010) to 25% (Murray et al., 2008).

Sorghum is the most closely related species to sugarcane, with a fully sequenced genome and a large amount of available QTL data. Therefore, this species was used as a reference for selecting a region involved in an important trait, i.e., sugar accumulation, and identifying a homologous set of these genes in sugarcane. If these homologous genes diverged from sorghum after a speciation event and if they came from the same duplicated group, then they are orthologs (Fitch, 1970) and should have the same function in sugarcane and sorghum. However, further investigation is needed to check if there is evidence of QTLs in this region that are associated with sugar accumulation in sugarcane. This approach, “targeted sequencing by gene synteny,” was possible once nearly all the genes were found in the sugarcane transcriptome, as described by Cardoso-Silva et al. (2014). A total of seven genes were not detected in the transcriptome described by Cardoso-Silva et al. (2014); four of these genes were found in a more recently published transcriptome described by Mattiello et al. (2015), and two of these transcripts were shared in a transcriptome described by Hoang et al. (2017). These results showed a high level of synteny in sorghum. More than 100,000 SP80-3280 BAC clones were used as a resource to access the sugarcane genome and recover this complex region. The positive BAC clones for two or more markers were selected for sequencing. A double selection strategy avoided small duplicated regions, pseudogenes and transposable elements carrying gene fragments as well as dramatically reduced the number of BAC clones selected. The advent of third-generation sequencing, and especially technologies resulting in the longest read lengths, such as single molecule real-time (SMRT) DNA sequencing (Eid et al., 2009), may facilitate the assembly process for segmental duplication problems caused by repetitive elements in complex genomes (English et al., 2012).

Large inserts of repetitive elements were observed between genes, but few large repetitive sequences were observed in intron sequences. Such large repetitive sequences in introns have been previously reported in other plants and do not necessarily affect the function of the gene (Kim and Zilberman, 2014). The high level of collinearity between the sorghum and sugarcane genes was utilized to identify the sugarcane homeologous regions associated with the absence of collinearity for repetitive regions (Jannoo et al., 2007; Garsmeur et al., 2011).

According to the comparative analysis with sorghum, at least 1.25 Mb, which was represented by nine sugarcane BAC sequences, was necessary to provide almost total coverage of the QTL region employing the “targeted sequencing by gene synteny” strategy. Some BAC sequences showed overlapping potential clustering in four syntenic blocks, with a highly conserved level of gene collinearity. For BAC clones that showed synteny with sorghum regions, there were two possibilities: complete overlap between BAC sequences suggested that the BACs came from the same homeologous chromosome, whereas total gene collinearity between BAC sequences and unaligned intergenic regions suggested that the BAC sequences came from different homeologous chromosomes. The choice of sorghum QTL stable and rich genes enabled these results to represent estimates for a small region of the sugarcane genome, ensuring the non-randomness of the results. Six of these sugarcane genes presented tandem duplications and could be attributed to the whole-genome duplication and polyploidization process (Alix et al., 2017). Additionally, these genes were inserted in an important biological region for sugarcane, and some hypotheses can be put forward to explain how these genes have maintained their original functions: if the original locus is disabled by mutation, the second gene can supply the necessary functional redundancy, or if both copies are maintained, they could increase the production of a gene product (Ohno, 1970).

These results represent an important step in understanding the genome structure of sugarcane and elucidating the complex architecture of the genomic region. This region should be associated with sugar accumulation. In addition, we propose a sequencing strategy for genome studies in polyploid species or diploid species originating via polyploidization, which present a huge challenge for obtaining the whole-genome sequence. The “targeted sequencing by gene synteny” approach can be applied to such species with complex genomes, especially those that have closely related diploid species with sequenced whole genomes. Furthermore, the use of BACs represents a powerful tool for recovering loci linked to important traits and determining homeologous regions associated with specific loci. Adding syntenic information to sequencing of non-random genome regions enables improving our understanding of genetic structure and identifying molecular markers physically linked to genes of interest in complex species. This strategy is very efficient and useful for the sequencing of regions enriched in genes. These advantages may allow important applications of sequencing results in plant breeding programs of polyploid species, particularly if the whole-genome sequence is not yet available for the species of interest.

## Data access

All the assembled and annotated BAC sequences were deposited in NCBI GenBank under accession numbers MF737006 to MF737073, and each sequencing pool was deposited in NCBI GenBank under SRA numbers SRR6760342 to SRR6760350. All the data can be found under Bioproject PRJNA398673.

## Author contributions

MM and DS: Conducted the experiments; CC-S: Analyzed the sequencing data; MM, CC-S, DS, and AP: Wrote the manuscript. All authors discussed the data, interpreted the results, read and edited the manuscript and approved the final version.

### Conflict of interest statement

The authors declare that the research was conducted in the absence of any commercial or financial relationships that could be construed as a potential conflict of interest.

## Supplementary material

Data Sheet S1 This file contains Supplementary Information including *in silico* data source, BAC screening, assembly and annotation description.

Data Sheet S2 File containing the FASTA format sequence of the sorghum target region.

Table S1 BLAST results for the sugarcane transcript selection.

Table S2 Primers used to screen the sugarcane BAC library.

Table S3 Distribution of the predicted transposable elements among the annotated BAC sequences.

Table S4 Summary of the sugarcane predicted gene annotation and alignment against sorghum, maize and rice proteomes.

Figure S1 Number of reads mapped onto non-annotated BAC contigs.

Figure S2 Lengths of the sugarcane non-annotated contigs representing the lowest contigs from each pool.

Figure S3 Distribution of the number of reads mapped onto each annotated BAC sequence.

Figure S4 Contig-length distribution of the sugarcane annotated BAC sequences.
