# Supplementary material for: “Targeted Sequencing by Gene Synteny,” a New Strategy for Polyploid Species: Sequencing and Physical Structure of a Complex Sugarcane Region
Source: Front Plant Sci. 2018 Mar 28;9:397. doi: 10.3389/fpls.2018.00397 (PMC5882829; doi:10.3389/fpls.2018.00397)
Supplement: Data Sheet S1 — This file contains Supplementary Information including in silico data source, BAC screening, assembly and annotation description. [file DataSheet1.docx]

**“Targeted Sequencing by gene synteny,” a new strategy for polyploid species: Sequencing and physical structure of a complex sugarcane region**

Melina Cristina Mancini^1§^, Claudio Benicio Cardoso-Silva^1§^, Danilo Augusto Sforça^1^, Anete Pereira de Souza^1,2^

^1^Center for Molecular Biology and Genetic Engineering (CBMEG), University of Campinas (UNICAMP), Campinas, SP, Brazil, ^2^Departamento de Biologia Vegetal, Instituto de Biologia, Universidade Estadual de Campinas (UNICAMP), Campinas, SP, Brazil

e-mail: anete@unicamp.br

**Supplementary Information**

***In silico* data sources (sorghum and sugarcane)**

More detailed information about the target QTL mapped by Murray et al. (2008) was obtained based on the sorghum consensus map reported by Mace et al. (2009), allowing us to anchor the QTL with flanking molecular markers. Murray et al (2008) used an inbred population from a cross between a high-biomass sweet sorghum variety and an elite inbred grain sorghum variety. QTL detection was performed via composite interval mapping (CIM). This study was chosen due to its mapping of QTLs in two different locations and years, indicating probable QTL stability, which is essential for non-randomness of detection. The authors identified QTLs for Brix on sorghum chromosomes 3, 6 and 7, among which chromosome 3 (SB-03) presented the same QTL mapped at two evaluated locations. Despite another trait related to sugar content being mapped to the same region, a higher proportion of phenotypic variation was explained by the genotype (R^2^ = 0.25 and 0.12 for QTL1 and QTL2, respectively) and LOD score (10 and 5.5 for QTL1 and QTL2, respectively). SB-03 is a region of the sorghum genome that has been extensively explored, as it confers resistance to drought (publication number: WO2013071366 A1) and aluminum tolerance (Magalhães et al., 2007). These previous studies provided the main motivation for investigating this region in the sugarcane genome.

The chosen QTL region was composed of approximately 2,120 kb in sorghum genome v3.1 available on Phytozome 12 database; starting at 55,205 kb and ending at 57,325 kb on SB-03. In total, it comprised 14 markers, including seven microsatellites (dominance marker; txp31, txp336, txp183, mSbCIR276, txp444, txp503, txp120), five RAPD/AFLP/DArTs (codominance marker; sPb1700, sPb8787, txs584, txS1439, txS536) and two genes (umc93, umc63). Within this region, the portion that was most saturated with dominance markers was selected for this study, between the “txp31” and “txp444” markers, resulting in a region of the sorghum genome of approximately 700 kb in length. Thus, the target region began at 55,265 kb and ended at 55,952 kb on SB-03 (Data Sheet S2).

This interval was employed to search for sorghum genes. The sequences of each molecular marker in this region were used to locate the exact chromosome position in the sorghum genome deposited in the Phytozome database (http://www.phytozome.net/) and to determine the relevant genes (introns and exons). In total, 60 sorghum genes were identified. The exon sequences were aligned against the sugarcane leaf transcriptome described by Cardoso-Silva et al. (2014) using BLASTn (Altschul et al., 1990) with an e-value cutoff of 1e-10. Of the 60 sorghum genes, 54 were highly similar (identity ≥ 90%) to sugarcane transcripts (Table S1). These sugarcane transcripts with a high identity to sorghum genes were selected for the development of specific primers for qPCR genotyping by Integrated DNA Technologies (Coralville, Iowa, USA).

All of the 54 markers developed for sugarcane exhibited annealing temperatures of 56 or 60°C using SP80-3280 genomic DNA. qPCR amplification was performed in a final volume of 5 µl, containing 2 ng of DNA, 0.16 μM each primer and 1X SYBR Green Master Mix (BIO-RAD). The cycling parameters employed for amplification were as follows: 95°C for 8 min for initial denaturation, then 50 cycles of 95°C for 20 sec and 56 or 60°C for 40 sec, finishing with a temperature ramp from 65 to 95° increasing at 0.5°/cycle for melting curve analysis. There were 18 markers that annealed at 56°C, 28 markers that annealed at 60°C, and eight markers that did not result in amplification (Table S2). Twelve of the 54 primer pairs tested on sugarcane genomic DNA resulted in no or poor amplification.

**BAC library screening**

The BAC library used in this study was constructed for the Brazilian hybrid sugarcane cultivar SP80-3280. The library is available at INRA (<http://cnrgv.toulouse.inra.fr/library/genomic_resource/Shy-B-80_3280>) and can be considered the largest sugarcane BAC library, with a total of 222,184 clones and an average insert size of 110 kb, covering the total sugarcane genome at 2.4X based on an estimated whole-genome size of 10 Gb (D’Hont and Glaszmann, 2001). Half of these clones were organized in 12 3D pools (3-Dimensional pools). Each 3D pool was reorganized into 12 ‘superpools’. Screening of the ‘superpools’ and 3D pools allowed direct identification of positive clones. The 3D pool screening approach dramatically reduced the time necessary for gene selection (Farrar and Iain, 2007).

Screening started with the 12 ‘superpools’, following the qPCR amplification conditions outlined above. Based on comparisons between the melting curves for each ‘superpool’, marker and SP80-3280 genomic DNA, a total of 348 positive 3D pools were selected. Through screening of the 3D pools (amplification conditions outlined above), more than 5,000 positive BAC clones were found. Positive fragments were separated via electrophoresis in a 2% agarose gel in 0.5X TBE to confirm the expected fragment size, based on a 1 kb lambda molecular weight marker.

The markers Sh17, Sh34, Sh39 and Sh50 exhibited nonspecific clone selection during library screening (they selected many or almost all clones) and were removed from screening. To avoid false positives, the double selection approach was employed. In this approach, more than 600 positive BAC clones were selected for at least two positive genes. The positive BAC clones were organized into five groups based on gene similarity. The BAC clones that were positive for the same genes were considered similar and were eliminated from the selection, decreasing the number to 118 clones. The 118 positive clones were separated for validation for the specific gene group. The clones considered false positives and those that were similar to other clones were removed. In total, 68 BACs were selected for sequencing using the PacBio platform. Each gene was represented by at least two BACs, ensuring target region coverage. Samples were pooled ensuring that each similar clone was located in a different sequencing run, utilizing a total of nine SMRT sequencing cells.

BAC DNA from the 68 clones was isolated using the Nucleobond Xtra Midi Plus (Macherey-Nagel, Düren, Germany) following the manufacturer’s instructions, with 50 mL LB media and a chloramphenicol selective marker. The quantity and quality of the BAC DNA was checked using a 1% agarose gel with Lambda HindIII as the standard and was verified using the 260/280 ratio by NanoDrop (ThermoFisher, Massachusetts, USA). A total of 20 µg BAC DNA per pool was dried using the speed vacuum.

**BAC assembly**

Unlike other investigations involving sequencing of BAC pools and hybrid assembly using second- and third-generation sequencing platforms (Huddleston et al., 2014; Setta et al., 2014; Okura et al., 2016), our study was performed using only PacBio data. To minimize PacBio errors, sequencing was performed with high coverage and an error correction pipeline was applied. Additionally, each BAC pool was assembled separately. As a result, high-quality sequences were obtained, with correctly assembled gene structures and BAC sequences presenting high synteny with the sorghum genome.

All the 180 contigs from the assembly with a size less than 50 Kb (Figure S1) were analyzed for the number of reads. Contigs with very low coverage (Figure S2) or less than five reads were eliminated from the analysis because they could represent reads with too many errors that were assembled together (represented by green in Figure S1). The remaining contigs were used to search for the genes expected in each region but contained no genes. BLASTn was also performed with the large-sized BACs, but no hits were returned. Therefore, the contigs with a size less than 50 Kb and high coverage (red colored in Figure S1) were excluded from further analyses. They may have represented fragments (intergenic regions) of the BACs that were not assembled into the larger contigs (Pool 01, Pool 02, Pool 07, Pool 08 and Pool 09) or small BACs that were not assembled (pool03 and pool04).

In total, 68 contigs were used in the subsequent analysis. These contigs showed high coverage (Figure S3) and were larger than 50 Kb (Figure S4). Pool03 and Pool04 resulted in one BAC less than what was expected, which may have caused an error when quantifying the BAC by inserting a small quantity of the DNA BAC into the pool, resulting in a low amount of DNA for sequencing. This may be the result of a BAC with a complex region, such as several repetitive elements that even the long PacBio reads could recover, resulting in small contigs that were eliminated based on their size (<50 Kb). Pool 05 and Pool 09 displayed an additional contig for each pool, which could be the result of a BAC that was assembled into two contigs due to a complex and/or repetitive region.

**BAC annotation and synteny analysis**

Sugarcane gene annotation was performed using the sorghum genome annotation as a reference (Sb v3.1). In addition, BLASTp was used to align predicted sugarcane genes against the proteomes of sorghum (*S. bicolor* v3.1), maize (*Z. mays* 2010-01) and rice (*O. sativa* 323 v7.0); these proteomes are available at Phytozome (https://phytozome.jgi.doe.gov/pz/ portal.html).

The annotation of repetitive elements (Table S3) resulted in 2 (Shy3280Sca060) to 167 (Shy3280Sca007) repetitive element fragments per BAC contig. Gene annotation (Table S4) using 68 BAC contigs resulted in 55 BAC sequences with at least one gene (Shy3280Sca018, Shy3280Sca023, Shy3280Sca026, Shy3280Sca029, Shy3280Sca035, Shy3280Sca036, Shy3280Sca038, Shy3280Sca039, Shy3280Sca043, Shy3280Sca048 and Shy3280Sca053) and one BAC sequence with 13 genes (Shy3280Sca040). No genes were found to be associated with thirteen BAC contigs.

According with Figure 02 in the manuscript, 20 BAC sequences were mapped to the corresponding sorghum gene positions. Interestingly, shy3280sca001 was the only BAC sequence without a corresponding homeologous sequence, whereas shy3280sca002 exhibited two corresponding homeologous sequences (shy3280sca018 and shy3280sca015). One gene (Sobic.003G218400) was found to exhibit tandem duplication in shy3280sca015, and shy3280sca003 was found to have four corresponding homeologous sequences (shy3280sca004, shy3280sca005, shy3280sca017 and shy3280sca016). One tandem duplication gene was identified in both shy3280sca004 and shy3280sca017 (Sobic.003G219700 and Sobic.003G219500, respectively), and two corresponding homeologous sequences were recovered for shy3280sca006 (shy3280sca020 and shy3280sca019). Two duplications were found in shy3280sca019 (Sobic.003G220600 and Sobic.003G220800), and shy3280sca007 presented one corresponding homeologous sequence (shy3280sca008), whereas shy3280sca009 had five corresponding homeologous sequences (shy3280sca012, shy3280sca010, shy3280sca014, shy3280sca013, shy3280sca011). One tandem duplication was found in shy3280sca014 (Sobic.003G222800).

**References**

Altschul, S.F., Gish, W., Miller, W., Myers, E.W., Lipman, D.J. (1990). Basic local alignment search tool. J Mol Biol. 215, 403-10.

Cardoso-Silva, C.B., Costa, E.A., Mancini, M.C., Balsalobre, T.W.A., Canesin, L.E.C., Pinto, L.R., et al. (2014). *De Novo* Assembly and Transcriptome Analysis of Contrasting Sugarcane Varieties. PLoS ONE **9**: e88462. doi:10.1371/journal.pone.0088462.

D’Hont, A., Glaszmann, J.C. (2001). Sugarcane genome analysis with molecular markers, a first decade research. Proc IntSoc Sugarcane Technol. 24, 556-559.

Farrar, K., and Donnison, I.S. (2007). Construction and screening of BAC libraries made from Brachypodium genomic DNA. Nature Protocols. 2,1661-74.

Huddleston, J., Ranade, S., Malig, M., Antonacci, F., Chaisson, M., Hon, L., et al. (2014). Reconstructing complex regions of genomes using long-read sequencing technology. Genome Res. 24, 688–696.

Mace, E.S., and Jordan, D.R. (2011). Integrating sorghum whole genome sequence information with a compendium of sorghum QTL studies reveals uneven distribution of QTL and of gene-rich regions with significant implications for crop improvement. Theor Appl Genet. 123, 169–191.

Magalhaes, J.V., Liu, J., Guimarães, C.T., Lana, U.G.P., Alves, V.M.C., Wang, Y.H., et al. (2004). A gene in the multidrug and toxic compound extrusion (MATE) family confers aluminum tolerance in sorghum. Nature Genetics. **39**(9): 1156-61. doi: 10.1038/ng2074.

Murray, S.C., Sharma, A., Rooney, W.L., Klein, P.E., Mullet, J.E., Mitchell, S.E., et al. (2008) Genetic improvement of Sorghum as a biofuel feedstock: I. QTL for stem sugar and grain nonstructural carbohydrates. Crop Science. 48. 2165-2179.

Okura, V.K., Souza, R.S.C., Tada, S.F.S., Arruda, P. (2016). BAC-Pool sequencing and assembly of 19 Mb of the complex sugarcane genome. Front Plant Sci. 7, 1-8.

Setta, N., Monteiro-Vitorello, C.B., Metcalfe, C.J., Cruz, G.M.Q., Del Bem, L.E., Vicentini, R., et al. (2014). Building the sugarcane genome for biotechnology and identifying evolutionary trends. BMC Genomics. **15**:540. doi: 10.1186/1471-2164-15-540. doi: 10.1186/1471-2164-15-540.
