# Supplementary figures and images for: “Targeted Sequencing by Gene Synteny,” a New Strategy for Polyploid Species: Sequencing and Physical Structure of a Complex Sugarcane Region
Source: Front Plant Sci. 2018 Mar 28;9:397. doi: 10.3389/fpls.2018.00397 (PMC5882829; doi:10.3389/fpls.2018.00397)

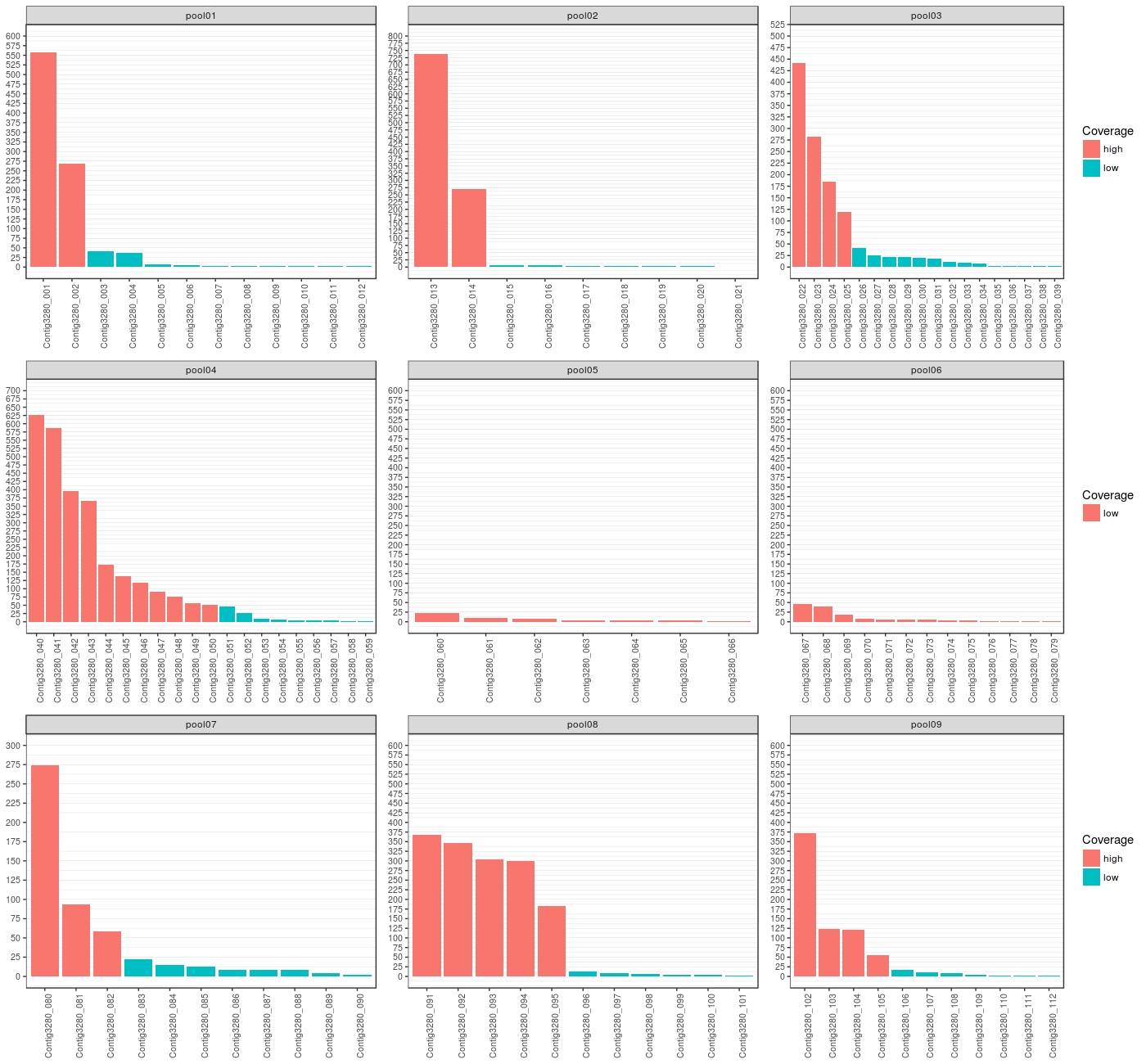

Supplement: Figure S1 — Number of reads mapped onto non-annotated BAC contigs. [file Image1.JPEG]

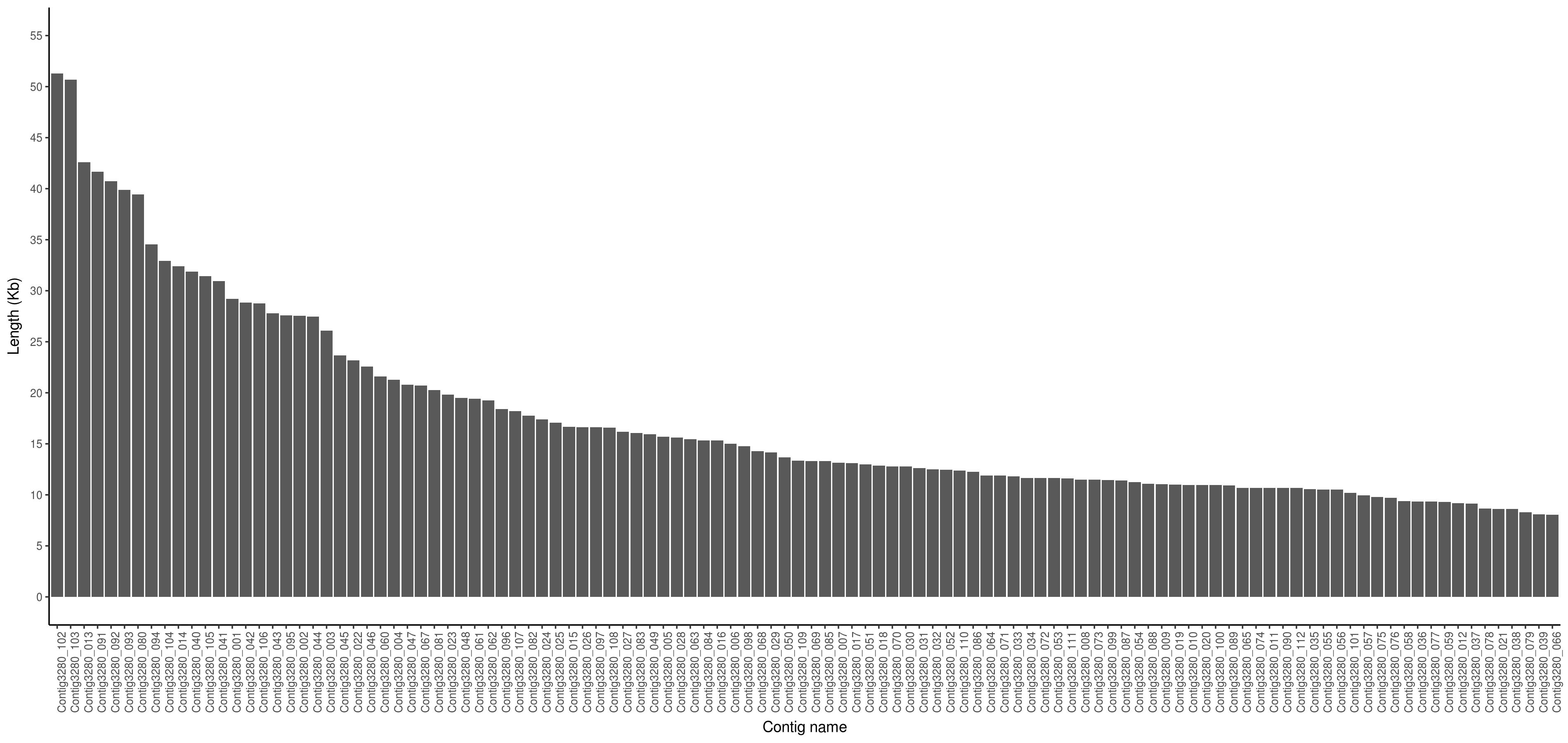

Supplement: Figure S2 — Lengths of the sugarcane non-annotated contigs representing the lowest contigs from each pool. [file Image2.JPEG]

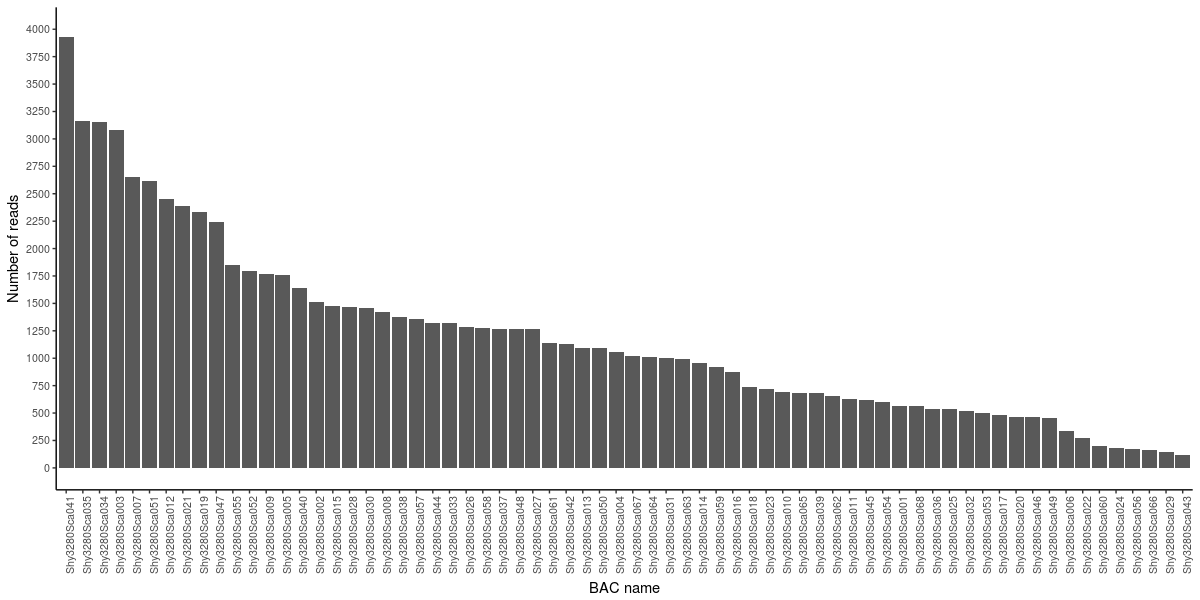

Supplement: Figure S3 — Distribution of the number of reads mapped onto each annotated BAC sequence. [file Image3.PNG]

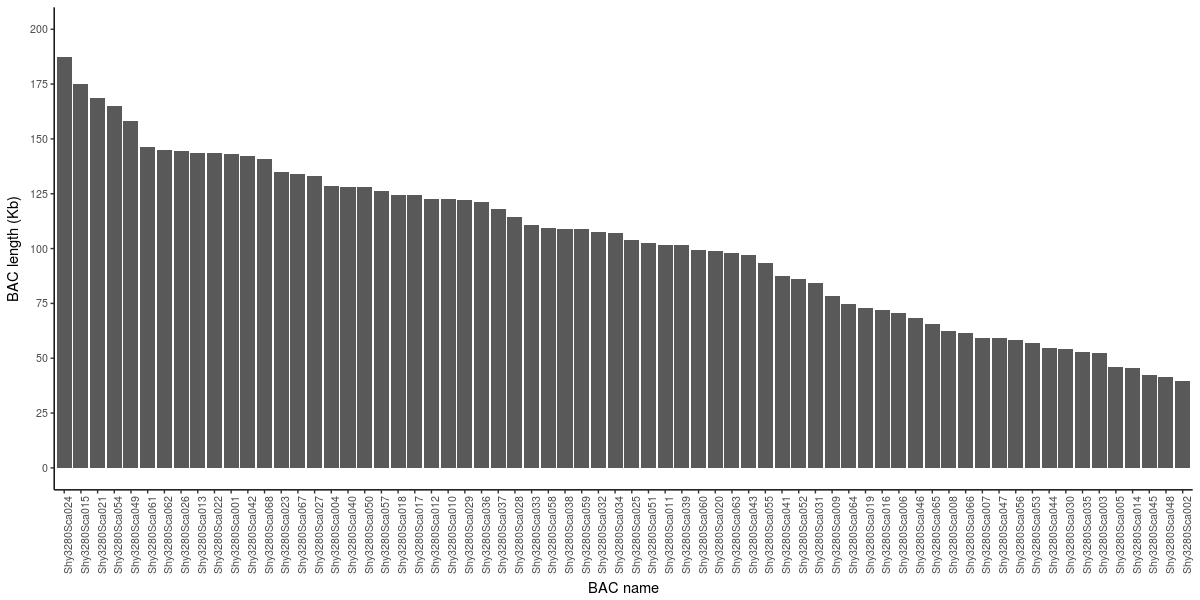

Supplement: Figure S4 — Contig-length distribution of the sugarcane annotated BAC sequences. [file Image4.PNG]
